# Acute exacerbation of chronic fibrosing interstitial pneumonia in patients receiving antifibrotic agents: incidence and risk factors from real-world experience

**DOI:** 10.1186/s12890-019-0880-0

**Published:** 2019-06-25

**Authors:** Kodai Kawamura, Kazuya Ichikado, Hidenori Ichiyasu, Keisuke Anan, Yuko Yasuda, Moritaka Suga, Takuro Sakagami

**Affiliations:** 1 Division of Respiratory Medicine, Social Welfare Organization Saiseikai Imperial Gift Foundation, Inc., Saiseikai Kumamoto Hospital, Chikami 5-3-1, Minami-ku, Kumamoto, 861-4193 Japan; 2 Department of Respiratory Medicine, Kumamoto University Hospital, Faculty of Life Sciences, Kumamoto University, Kumamoto, Japan

**Keywords:** IPF, Antifibrotic agent, Corticosteroids, Chronic fibrotic interstitial pneumonia, Proton pump inhibitor

## Abstract

**Background and objective:**

Here, we present real-world data on the incidence and risk factors of acute exacerbation (AE) in patients with chronic fibrotic interstitial pneumonia (CFIP) treated with antifibrotic agents, which has been previously poorly documented.

**Methods:**

We retrospectively examined clinical characteristics, incidence and risk factors of AE in a cohort of 100 patients with CFIP (*n* = 75, idiopathic pulmonary fibrosis [IPF]; *n* = 25, other conditions), all of whom received antifibrotic agents in a real-world setting.

**Results:**

The median follow-up was 17.4 months (interquartile range [IQR], 6.6 to 26.7 months). During the follow-up periods, 21 patients experienced AE after starting antifibrotic agents. The estimated 1-, 2-, and 3-year AE incidence rates were 11.4% (95% confidence interval [95%CI], 6.2–20.3%), 32% (95%CI, 20.7–47.4%), and 36.3% (95%CI 23.5–53.1%), respectively. Decreased baseline lung function (forced vital capacity and carbon monoxide diffusing capacity of the lung), existence of pulmonary hypertension estimated from an echocardiogram, higher *Interstitial Lung Disease-*Gender, Age, and Physiology (ILD-GAP) score, supplementary oxygen, and concomitant corticosteroid and proton-pump inhibitor (PPI) use upon starting the antifibrotic agent were risk factors of AE. Concomitant corticosteroid and PPI use and corticosteroid dose were risk factor of AE in a multivariate Cox regression hazard model adjusting for ILD-GAP score.

**Conclusion:**

AE of CFIP is more common in patients with physiologically and functionally advanced disease under antifibrotic agents. Prudent use of corticosteroids and PPIs when initiating antifibrotic agents may be recommended. Further studies are warranted.

**Electronic supplementary material:**

The online version of this article (10.1186/s12890-019-0880-0) contains supplementary material, which is available to authorized users.

## Introduction

Chronic fibrotic interstitial pneumonia (CFIP) is associated with substantially reduced health-related quality of life and survival. Idiopathic pulmonary fibrosis (IPF) is a type of CFIP. The disease course of progressive CFIPs, such as IPF, is variable and unpredictable; however, the median survival time after IPF diagnosis is 3–5 years [1–3].

Until recently, no effective pharmacological options existed for IPF; however, two drugs are now approved for antifibrotic therapy: pirfenidone [4] and nintedanib [5]. Antifibrotic therapy can slow declining lung function [5, 6] and reduce the risk of death from IPF [6–8], but it cannot stop the disease course. International guidelines recommend using antifibrotic agents for IPF patients [9].

Confidently diagnosing CFIP is challenging [10]. Recent international clinical guidelines recommend performing surgical lung biopsies when physicians cannot confidently diagnose IPF [11]. However, in real-world practice, performing surgical lung biopsies and bronchoscopies can be difficult because of age, advanced disease, or patient refusal. In these cases, physicians sometimes prescribe antifibrotic agents when computed tomography imaging and pulmonary function tests show fibrotic processes in the lungs indicating that IPF is likely. Clinical trials evaluating antifibrotic agent efficacy and safety in patients with progressive fibrosing lung diseases other than IPF are ongoing [12, 13].

Most IPF patients have relatively slow clinical courses, but some experience acute respiratory worsening, namely acute exacerbation (AE) [14]. A recent epidemiological survey of Japanese patients with IPF revealed that the most common cause of death was AE-IPF [2]. Several studies found that AE occurs in other CFIPs as well [15, 16].

Previous studies found that AE-IPF is more common in patients with physiologically and functionally advanced disease [14]. Recent clinical trial data on AE incidence among patients treated with antifibrotics revealed that antifibrotics may reduce the probability of AE-IPF [5, 17]. However, real-world data on AE incidence and risk factors among patients treated with antifibrotics are poorly documented. Most data on AE risk factors are derived from the pre-antifibrotic agent era; thus, the risk factors for AE-CFIP in patients treated with antifibrotic agents are not well known. To address these clinical questions, this study determined the AE incidence and risk factors in CFIP patients treated with antifibrotic agents in a real-world clinical setting.

## Methods

The institutional review board of Saiseikai Kumamoto Hospital approved this study, which was conducted in accordance with the Declaration of Helsinki. Because the study was retrospective, informed consent from participants was unrequired per the “Ethical Guidelines for Medical and Health Research Involving Human Subjects” presented by the Japanese Ministry of Health, Labour and Welfare.

### Patients

Patients with progressive CFIP treated with antifibrotic agents (pirfenidone or nintedanib) from 01 Aug 2015 to 31 Aug 2018 with no history of AE were identified at Saiseikai Kumamoto Hospital. All patients received antifibrotic agents as the standard-of-care. Follow-up included an inpatient visit or phone call by a research team member. Date of last follow-up was identified as either date of death, last in-person visit or last research phone call. Data were locked on 30 Sept 2018. Subjects were censored if they [1] experienced no events by 30 Sept 2018 or [2] were lost to follow-up.

Demographic, clinical, and pathological data were collected from electronic medical records. Patients’ pharmacy records were reviewed to determine whether patients were documented as having received corticosteroids, anticoagulant and/or antiplatelet drugs, and H2-blockers or proton-pump inhibitors upon initiating the antifibrotic agent. Corticosteroid dose was expressed as total daily milligrams of prednisone equivalents. The following clinical characteristics were collected for all patients: age upon antifibrotic initiation, gender, time from 1st visit to antifibrotic initiation (months), surgical lung biopsy (yes or no), clinical diagnosis upon starting antifibrotic agents (IPF vs others: chronic hypersensitivity pneumonitis [CHP], collagen vascular disease-associated IP, and unclassifiable), long-term oxygen therapy (no, therapy started prior to antifibrotic agent initiation, or therapy introduced simultaneously with antifibrotic agents), serum KL-6 level, serum lactate dehydrogenase (LDH), *Interstitial Lung Disease-*Gender, Age, and Physiology (ILD-GAP) score [18], smoking history, and updated Charlson comorbidity index [19]. Baseline pulmonary function testing, right ventricular systolic pressure estimated by echocardiogram (> or ≤ 40 mmHg), serum KL-6, and serum LDH levels at baseline were performed on the day the antifibrotic agent was initiated or within 14 days before the first antifibrotic agent treatment. ILD-GAP scores were calculated from data obtained at the start of antifibrotic agent use. Using the ILD-GAP index, we classified patients into one of three categories based on enrollment values: stage 1 (0–3 points), stage 2 (4–5 points), and stage 3 (6–8 points).

Diagnoses of acute exacerbations of interstitial pneumonia were made in accordance with the definition of acute exacerbation of IPF proposed by Collard et al. [14], which includes both triggered and idiopathic acute exacerbations. IPF was diagnosed by the diagnostic criteria proposed by the American Thoracic Society, European Respiratory Society, Japanese Respiratory Society, and Latin American Thoracic Association [20] and also included cases confirmed to have features of possible usual interstitial pneumonia (UIP) and traction bronchiectasis on high-resolution computed tomography with no surgical lung biopsy as previously described [21]. Non-IPF was diagnosed after discussing and integrating the clinical, radiological, bronchoalveolar lavage, and pathological findings.

### Statistical analysis

Patients’ baseline characteristics were summarized using percentages for categorical variables and medians and interquartile ranges for continuous variables. Between-group comparisons were performed using the Mann-Whitney rank-sum and Fisher’s exact tests. Time-to-event endpoints were defined as the time from antifibrotic initiation to the first admission of AE. Time-to-end endpoints were estimated using the Kaplan-Meier method based on events and compared with the log-rank test (univariable analysis). Risk factors for time to first AE development and univariate survival were analyzed using the Cox proportional hazards model to determine hazard ratios (HR) and 95% confidence intervals (95%CI). For concomitant medications, multivariable logistic regression was performed for AE risk factors using an a priori covariable of ILD-GAP scores in some covariate.

We used the inverse probability of treatment-weighting analysis using the propensity score calculated from the data at the start of treatment to analyze the pharmacological treatment effect for the AE risk as a sensitivity analysis. All tests were two-sided and performed at a significance level of 0.05. All statistical analyses were performed using EZR (Saitama Medical Center, Jichi Medical University, Saitama, Japan) [22], a graphical user interface for R, version 3.2.2 (The R Foundation for Statistical Computing, Vienna, Austria).

## Results

### Baseline characteristics

One hundred patients treated with antifibrotic agents without previous history of AE were recruited. Three patients who started antifibrotics after AE were identified and excluded from this analysis.

Table 1 shows the patients’ baseline demographics and clinical characteristics. The study cohort included 75 men and 25 women, and the patients’ median age was 68 years (interquartile range [IQR] 64–73 years). The median follow-up was 17.4 months (IQR 6.6–6.7 months). The median time from first visit to starting antifibrotics was 12 months (IQR 6–36 months). The clinical diagnoses upon starting antifibrotics were IPF (*n* = 75) and others (*n* = 25; CHP = 19; collagen-vascular disease-associated [rheumatoid arthritis] =2; unclassified = 4). The baseline median values for percent predicted forced vital capacity (FVC) and percent predicted diffusing capacity of the lung for carbon monoxide (DLCO) were 70.8% (IQR 58.7–82.4%) and 55.9% (IQR 45.1–68.4%), respectively. Patients who had been diagnosed as not-IPF before commencing anti-fibrotic agents were more frequently prescribed corticosteroids than those with an IPF diagnosis at the time of starting anti-fibrotic agents (IPF *n* = 15 [20%] vs. non-IPF *n* = 11 [44%]; *p* = 0.033).

**Table 1:** Baseline characteristics of patients treated with antifibrotic agents

Factor	Group	Overall (*n* = 100)
Age		68 [64, 73]
Age ≥ 65 yrs. (%)	Yes	73 (73.0)
Sex (%)	Male	75 (75.0)
Smoking history (%)	Yes	69 (69.0)
Clinical diagnosis	IPF	75 (75)
	others	25 (25)
SLB (%)	Yes	29 (29.0)
Commodity index		1.0 [1.0, 1.0]
TTE RVSP	≥40 mmHg	29 (29.0)
FVC % predicted		70.8 [58.7, 82.4]
FVC % predicted < 70	Yes	48 (48.0)
DLCO % predicted		55.9 [45.1, 68.4]
DLCO % predicted < 55	Yes	45(45.0)
ILD-GAP score		3 [2, 4]
ILD-GAP score	0–3	61 (61.0)
	4–5	31 (31.0)
	6–8	8 (8.0)
LTOT	No use	63 (63.0)
	Introduce at same time	29 (29.0)
	Prior to antifibrotic agent	8 (8.0)
KL6		1265 [804, 2046]
LDH		239.5 [208, 265]
Time from diagnosis to antifibrotic agent (per month)		12 [6, 36]
Medication		
Antifibrotic agent	Nintedanib	52 (52.0)
	Pirfenidone	48 (48.0)
H2 blocker use	Yes	12 (12)
PPI use	Yes	62 (62)
Corticosteroid use	Yes	26 (26)
Corticosteroid dose	mg/body/day	0 [0, 5]
Corticosteroid dose in patients receiving corticosteroids	Mg/body/day	10[10, 20]
Anticoagulant (%)	Yes	18 (18.0)

No (%), median [IQR]

*IPF* idiopathic pulmonary fibrosis, *SLB* surgical lung biopsy, *TTE* transthoracic echocardiogram, *RVSP* right ventricular systolic pressure, *FVC* forced vital capacity, *DLCO* carbon monoxide diffusing capacity of the lungs, *ILD-GAP* interstitial lung disease subtype, gender, age, and two lung physiology variables, *LTOT* Long-term oxygen therapy, *PPI* proton pump inhibitor, *LDH* lactate dehydrogenase

### AE incidence

**Fig. 1:**
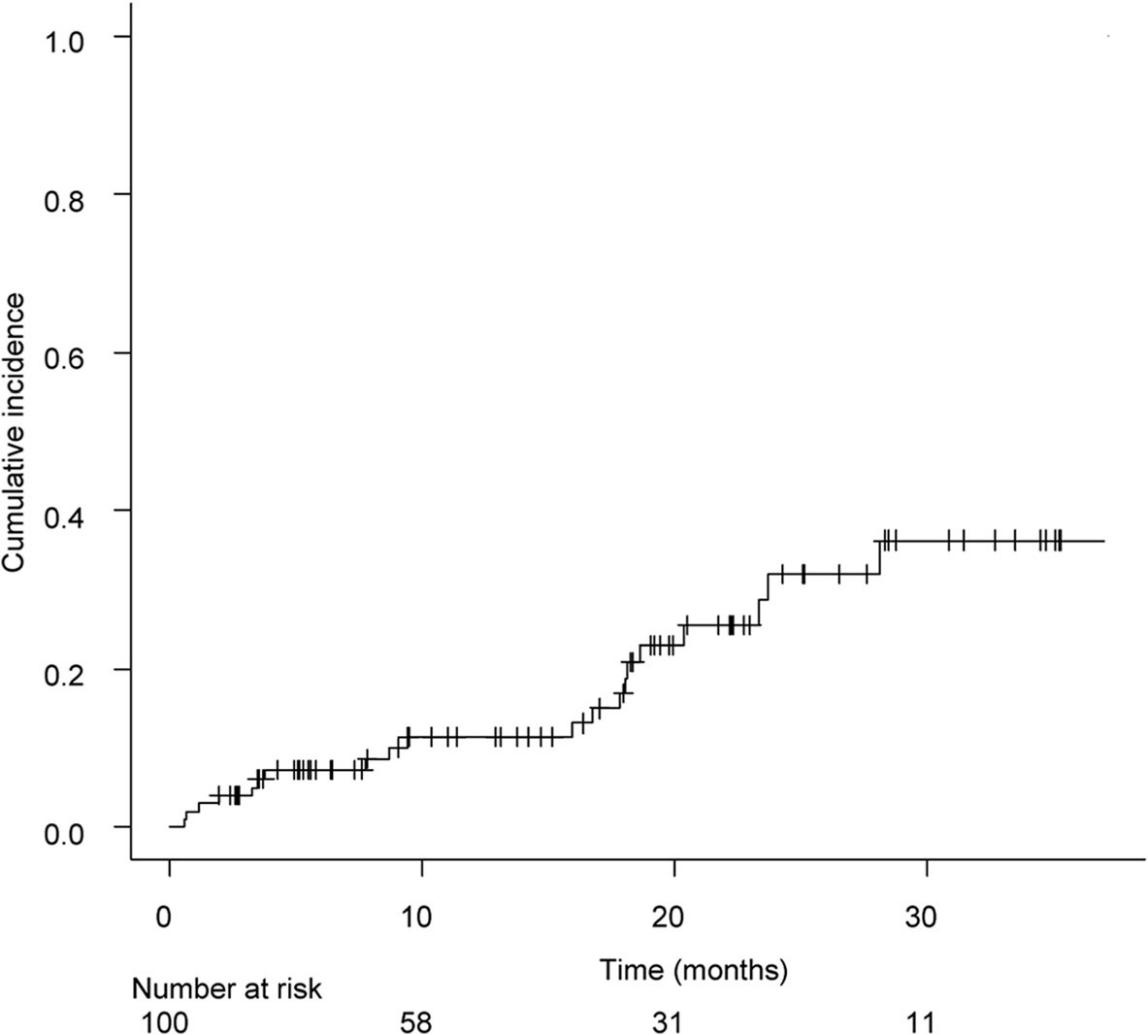
Cumulative incidence of acute exacerbation of chronic fibrotic interstitial pneumonia

During the follow-up periods, 21 patients experienced AE after antifibrotic agent introduction. Figure 1 shows the cumulative incidence of AE-CFIP. The estimated 1-, 2-, and 3-year AE incidences were 11.4% (95%CI, 6.2–20.3%), 32% (95%CI, 20.7–47,4%), and 36.3% (95%CI, 23.5–53.1%), respectively. Table 2 compares the baseline characteristics and patient outcomes with and without AE. Sex, smoking history, clinical diagnosis, and comorbidity index distributions did not significantly differ between groups. Patients with AE were slightly younger than those without AE, but the percentage of elderly patients did not differ between groups. There was a tendency for patients who developed AEs to have had SLBs (47.6 versus 24.1% *p* = 0.06); however, the difference was not statistically significant. No AEs were associated with surgical lung biopsy. Patients with AE had decreased lung functions; higher ILD-GAP scores; more frequent use of corticosteroids, PPIs, and long-term oxygen therapy; and longer times from diagnosis to starting antifibrotic agents than did the non-AE group.

**Table 2:** Comparison of baseline characteristics between patients with and without acute exacerbation

Factor	Group	Non-AE	AE (+)	*P*-value
		(*n* = 79)	(*n* = 21)	
Age	y	69 [65, 74]	66 [63, 68]	0.034
Age > 65 (%)	< 65	18 (22.8)	9 (42.9)	0.095
	≥65	61 (77.2)	12 (57.1)	
Sex (%)	Female	23 (29.1)	2 (9.5)	0.089
	Male	56 (70.9)	19 (90.5)	
Smoking history (%)	Yes	55 (69.6)	14 (66.7)	0.79
Clinical diagnosis upon staring antifibrotic agent	IPF	60 (75.9)	15 (71.4)	0.78
	others	19 (24.1)	6 (28.6)	
SLB (%)	Yes	19 (24.1)	10 (47.6)	0.06
Commodity. Index		1 [1, 1]	1 [1, 1]	0.32
TTE RVSP (%)	≥40 mmHg	19 (24.1)	10 (47.6)	0.06
FVC % predicted		75.5 [61.0, 83.4]	62.6 [52.6, 76.1]	0.03
FVC % predicted	< 70	34 (43.0)	14 (66.7)	0.08
DLCO % predicted		56.3 [48.9, 68.7]	44.80 [32.40, 64.30]	0.02
DLCO % predicted	< 55	32 (40.5)	13 (61.9)	0.09
ILD-GAP score		3 [2, 4]	4 [3, 5]	0.03
ILD-GAP score	0–3	51 (64.6)	10 (47.6)	0.02
	4–5	25 (31.6)	6 (28.6)	
	6–8	3 (3.8)	5 (23.8)	
LTOT	No	54 (68.4)	9 (42.9)	0.009
	Introduced at same time	22 (27.8)	7 (33.3)	
	Prior to antifibrotic agent	3 (3.8)	5 (23.8)	
KL6		1210 [795, 1985.5]	1530 [977, 2160]	0.37
KL6	Low	42 (53.2)	8 (38.1)	0.333
	High	37 (46.8)	13 (61.9)	
LDH		239 [208.5, 264.5]	242 [205, 305]	0.56
LDH high (%)	Low	40 (50.6)	10 (47.6)	1
	High	39 (49.4)	11 (52.4)	
Time from diagnosis to antifibrotic agent (per month)		12 [5, 25]	30 [12, 50]	0.02
Medication				
first-line antifibrotic agent	Nintedanib	43 (54.4)	9 (42.9)	0.46
	Pirfenidone	36 (45.6)	12 (57.1)	
H2 blocker use	No	70 (88.6)	18 (85.7)	0.71
	Yes	9 (11.4)	3 (14.3)	
PPI use	No	35 (44.3)	3 (14.3)	0.01
	Yes	44 (55.7)	18 (85.7)	
Corticosteroid use	Yes	12 (15.2)	14 (66.7)	< 0.001
Anticoagulant use	Yes	16 (20.3)	2 (9.5)	0.35

No (%), median [IQR]

*AE* acute exacerbation, *IPF* idiopathic pulmonary fibrosis, *SLB* surgical lung biopsy, *TTE* transthoracic echocardiogram, *RVSP* right ventricular systolic pressure, *FVC* forced vital capacity, *DLCO* carbon monoxide diffusing capacity of the lungs, *ILD-GAP* interstitial lung disease subtype, gender, age, and two lung physiology variables, *LTOT* Long-term oxygen therapy, *PPI* proton pump inhibitor, *LDH* lactate dehydrogenase

Additional file 1: **Table S1** lists details of each patient’s baseline characteristics. AE varied seasonally and appeared more frequently during winter.

### Risk factors for AE

**Fig. 2:**
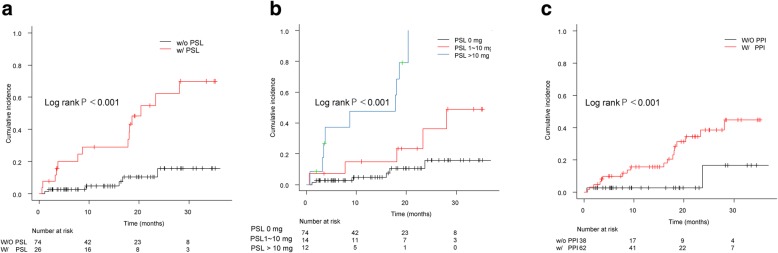
(**a**) Cumulative incidence of acute exacerbation based on concomitant corticosteroid (with[w/] or without[w/o] PSL) use at antifibrotic agent initiation; (**b**) Cumulative incidence of acute exacerbation based on baseline corticosteroid dose at antifibrotic agent initiation; (**c**) Cumulative incidence of acute exacerbation based on concomitant use of proton-pump inhibitors (PPI)

Table 3 lists risk factors of AE. Decreased baseline lung function (FVC, DLCO), estimated right ventricular systolic pressure over 40 mmHg by echocardiogram, and higher ILD-GAP score and stage were risk of AE. Patients receiving long-term oxygen therapy prior to starting antifibrotics had higher risks of AE (HR 4.8; 95%CI 1.6–14.7; *P* = 0.006) than did patients who had not received prior oxygen therapy or patients who began both simultaneously. Patients receiving corticosteroids upon beginning antifibrotics had higher risks of AE (adjusted HR 11.3; 95%CI 4.1–32.0; *p* < 0.0001) than those not receiving corticosteroids, independent of underlying disease severity. Additionally, AE was increased in patients receiving > 10 mg or 1–10 mg prednisone compared with patients not receiving corticosteroids (Fig. 2b).

**Table 3:** Unadjusted and adjusted risk factors of acute exacerbation

		Hazard ratio	95%CI		*p*-value
Age		0.97	0.92	1.03	0.34
Age	< 65	ref			
	≥65	0.65	0.27	1.60	0.35
Sex	Female	ref			
	male	2.74	0.63	11.8	0.18
smoking	Yes	0.76	0.30	1.91	0.56
Clinical Diagnosis	IPF	Ref			
	others	0.83	0.3	2.28	0.71
SLB, yes	yes	2.11	0.88	5.08	0.10
Commodity Index		0.82	0.44	1.50	0.52
TTE RVSP 40 mmHg	≥40 mmHg	2.51	1.05	6.05	0.04
FVC % predicted		0.97	0.95	0.99	0.026
FVC % predictive < 70%	Yes	2.53	1.0	6.3	0.049
DLCO % predicted		0.95	0.92	0.98	0.002
DLCO % predicted < 55%	Yes	2.3	0.92	5.8	0.08
GAP score		1.55	1.18	2.05	0.002
ILD-GAP score	0–3 points	ref			
	4–5 points	1.44	0.51	4.08	0.50
	6–8 points	9.52	3.10	29.2	< 0.001
LTOT	No	ref			
	introduced at same time	2.01	0.73	5.55	0.18
	prior to antifibrotic agent	4.79	1.56	14.7	0.006
KL6		1	0.99	1	0.91
KL6 High	> 1300	1.54	0.63	3.78	0.35
LDH		1.001	0.999	1.002	0.19
LDH High	> 239	1.33	0.55	3.20	0.53
Medication					
H2 blocker use		0.70	0.16	3.04	0.63
PPI use		4.53	1.05	19.54	0.04
Corticosteroids		6.28	2.41	16.4	0.0002
anticoagulant	Yes	0.74	0.17	3.24	0.70
First-line antifibrotic agent	Nintedanib	Ref			
	Pirfenidone	0.98	0.40	2.39	0.96
Time from diagnosis to antifibrotic agents (per month)		1.01	1.004	1.029	0.008
Multivariate analysis		aHR	95%CI		*P*-value
H2 blocker use	Yes	0.44	0.099	1.92	0.27
PPI use	Yes	5.05	1.17	21.9	0.03
Corticosteroids	Yes	11.4	4.07	32.01	< 0.001
Anticoagulant	Yes	0.79	0.18	3.46	0.75
First-line antifibrotic agents	Nintedanib	Ref			
	Pirfenidone	1.26	0.50	3.20	0.62

*AE* acute exacerbation, *IPF* idiopathic pulmonary fibrosis, *SLB* surgical lung biopsy, *TTE* transthoracic echocardiogram, *RVSP* right ventricular systolic pressure, *FVC* forced vital capacity, *DLCO* carbon monoxide diffusing capacity of the lungs, *ILD-GAP* interstitial lung disease subtype, gender, age, and two lung physiology variables, *LTOT* Long-term oxygen therapy, *PPI* proton pump inhibitor, *LDH* lactate dehydrogenase

Patients receiving PPIs also had a greater risk of AE than those not on PPIs (Figure2c), independent of underlying disease severity (adjusted HR 5.1; 95%CI 1.2–21.9; *p* = 0.03). Concomitantly using H2 blockers and anticoagulant and/or antiplatelet drugs with antifibrotic agents was not an AE risk.

The AE incidence rates in patients on nintedanib and pirfenidone were 15.4% (95%CI 7.3–27.2%) and 17.4% (95%CI 9.3–28.4%) per 100 patient-years, respectively, but this was statistically insignificant (Incidence rate ratio 0.89; 95%CI 0.40–1.95; *P* = 0.76).

To analyze sensitivity, we performed the inverse probability of the treatment-weighting analysis using the propensity score calculated from the data taken upon starting antifibrotic treatment to analyze the pharmacological treatment effect on AE risk. In this analysis, corticosteroid and PPI use at baseline were a risk factor of AE in patients treated with antifibrotics. The adjusted HRs estimated using the propensity score were 4.2 (95%CI 1.4–13.3; *P* = 0.013) for corticosteroid use and 6.7 (95%CI 1.5–30.1; P = 0.013) for PPI use.

## Discussion

This study demonstrated the incidence and risk factors of acute exacerbation (AE) in patients with chronic fibrotic interstitial pneumonia (CFIP) treated with antifibrotic agents. AE-CFIP treated with antifibrotic agents was more common in patients with physiologically and functionally advanced diseases, as previously reported. Concomitantly using corticosteroids and PPIs may be a risk factor of AE in patients with CFIP treated with antifibrotics.

The estimated 1-, 2-, and 3-year AE incidence rates were 11.4, 32, and 36.3%, respectively (for CIs, see results section), which was consistent with a previous report that AE occurs in approximately 5–15% of IPF patients [23]. A recent clinical trial found that AE occurs in 5–10% of patients on nintedanib [17]. AE incidence in this study cohort may have been slightly higher than that of recent clinical trial data from patients treated with antifibrotics. However, previous cohort studies have also reported higher AE-IPF incidences than have clinical trials, possibly because the real-world data included patients with more advanced disease.

A previous report revealed that AE-IPF is more common in patients with physiologically and functionally advanced disease [14]. Our results also indicated that AE was more common in patients with advanced disease, even when treated with antifibrotics, and that the ILD-GAP model [18] could better predict AE-CFIP. The ILD-GAP model modified the GAP model [24] to apply to IPF and other interstitial lung diseases based on the following variables: interstitial lung disease (ILD) subtype, gender, age, and two lung physiological variables (FVC and DLCO) [18]. GAP models were previously reported as good predictors of AE-IPF [25, 26]. Our results suggest that the ILD-GAP model could be used as a simple screening tool to predict AE-CFIP patients treated with antifibrotics.

We demonstrated that using corticosteroids upon antifibrotic initiation is dose-dependently associated with increased AE risk. Antifibrotic treatment has been a significant advance for IPF patients. As these agents have become a standard of care, common practices that may affect the antifibrotic’s efficacy must be recognized and studied. Recent clinical guidelines recommend not using corticosteroids for IPF either alone or combined with other medications [20, 27]. Thus, corticosteroid use is being gradually reduced. However, recent reports have shown that approximately 20% of patients still receive corticosteroids [8, 28–30], even in the antifibrotic-agent era. This percentage is consistent with our study’s findings. Corticosteroids are commonly administered for various indications (e.g., decreased appetite, fatigue, cough). Analysis of the INPULSIS trials demonstrated that corticosteroid use at baseline did not influence nintedanib’s effectiveness or the rate of FVC decline [31]. However, our study demonstrated that corticosteroid use when simultaneously initiating antifibrotics is associated with increased AE risk even dose-dependently at low doses, and AE greatly impacted CFIP mortality. Our results suggest that prudent corticosteroid use when initiating antifibrotics may be warranted.

In our analysis, PPI use was associated with higher AE incidences after adjusting for disease severity. Previous retrospective studies revealed that antacid use was associated with a slower decline in FVC over time [32] and possibly fewer AEs in patients with IPF [33]. The 2015 treatment guidelines for IPF conditionally recommended antacid use for treating IPF rather than gastroesophageal reflux disease [9] based on the aforementioned studies. Multiple studies published on antacid therapy in IPF patients present different conclusions regarding whether antacid therapy is beneficial. A recent study suggested antacids do not benefit the IPF clinical course [34, 35], and another suggested that baseline antacid use was most strongly associated with AE risk among IPF patients treated with nintedanib [36]. Our results indicated that PPIs were associated with an increased AE risk, but H2 blockers were not. PPIs are reported to be associated with increased risks of adverse events (e.g., community-acquired pneumonia and cardiovascular events) and excess risk of death [37]. PPIs are also recognized as affecting the lung microbiome [38]. A recent study discovered a change in the respiratory microbiome during an AE-IPF [39]. Thus, clinical trials are urgently needed to prospectively evaluate the efficacy of PPIs in IFP in patients taking antifibrotics.

This study had some limitations. First, it was performed at a single center and included patients with varying fibrotic ILD, disease severities, and comorbidities. However, the cohort’s demographic features and lung functions were comparable to those of other studies, and systematic history collection, physical examinations and blood tests were performed on all patients upon initiating antifibrotics. Second, because the number of patients was relatively small, we could not perform multivariate analyses including many factors. Third, the study lacked a control group not taking antifibrotics. Fourth, the study lacked detailed information on the duration and intensity of corticosteroid use while taking antifibrotics. Time-dependent covariate analyses were impossible given the limited data.

## Conclusions

In conclusion, this study showed that even in patients treated with antifibrotics, acute exacerbation (AE) of chronic fibrotic interstitial pneumonia (CFIP) is more common in those with physiologically and functionally advanced disease. This study demonstrated the need for early diagnosis and early treatment choices in patients with CFIP. Corticosteroid use at baseline may be a risk factor for AE with antifibrotic treatment. Given this study’s limitations, these results should be further validated in a larger cohort. Prudent indication for concurrent corticosteroid and proton-pump inhibitor use when initiating antifibrotic agents is recommended. Further prospective data are needed to assess the impact of corticosteroid and PPI use on IPF treated with antifibrotics.

## Additional file

Additional file 1: **Table S1.** lists details of each patient’s baseline characteristics. AE varied seasonally and appeared more frequently during winter (DOCX 16 kb)

## Data Availability

All data generated or analysed during this study are included in this published article and its supplementary information files.

## Abbreviations

AE: Acute exacerbation
CFIP: Chronic fibrotic interstitial pneumonia
CI: Confidence interval
DLCO: Diffusing capacity of the lungs for carbon monoxide
FVC: Forced vital capacity
HR: Hazard ratios
IPF: Idiopathic pulmonary fibrosis
IQR: Interquartile range
PPI: Proton-pump inhibitor
