# Supplementary material for: Acute exacerbation of chronic fibrosing interstitial pneumonia in patients receiving antifibrotic agents: incidence and risk factors from real-world experience
Source: BMC Pulm Med. 2019 Jun 25;19:113. doi: 10.1186/s12890-019-0880-0 (PMC6593518; doi:10.1186/s12890-019-0880-0)
Supplement: Supplementary file 1 — Table S1. lists details of each patient’s baseline characteristics. AE varied seasonally and appeared more frequently during winter (DOCX 16 kb) [file 12890_2019_880_MOESM1_ESM.docx]

Supplementary tableS1

| No | Diagnosis | SLB | Smoking History | LTOT | %FVC | %DLCO | RVSP  >40 mmHg | AFA | Time to AE (day) | Antacid drug | PSL(mg) | Season |
| --- | --- | --- | --- | --- | --- | --- | --- | --- | --- | --- | --- | --- |
| 1 | Non-IPF | Y | P | NO | 97.1 | 61.5 | N | PFD | 855 | PPI | 10 | Mar |
| 2 | Non-IPF | Y | P | Same | 52.6 | 49.4 | N | PFD | 552 | PPI | 10 | Apr |
| 3 | IPF | N | N | NO | 73.6 | 72.7 | N | PFD | 549 | PPI | 25 | Dec |
| 4 | Non-IPF | N | P | NO | 61 | 77 | N | PFD | 1130 | H2 | 0 | Sep |
| 5 | IPF | N | P | NO | 76.1 | 71.8 | N | PFD | 286 | PPI | 0 | Mar |
| 6 | Non-IPF | N | P | NO | 66.5 | 64.3 | N | PFD | 542 | PPI | 20 | Oct |
| 7 | IPF | Y | P | NO | 67.5 | 37.6 | Y | PFD | 620 | PPI | 20 | Oct |
| 8 | Non-IPF | Y | N | Same | 50.4 | 32.4 | Y | PFD | 264 | PPI | 15 | Feb |
| 9 | IPF | N | P | Same | 80.8 | 65.8 | N | PFD | 99 | PPI | 20 | Apr |
| 10 | IPF | N | N | NO | 44.4 | 71.4 | N | PFD | 721 | H2 | 0 | Nov |
| 11 | IPF | Y | P | NO | 67.9 | 44.8 | N | PFD | 236 | PPI | 10 | May |
| 12 | IPF | N | P | NO | 55.8 | 39.9 | N | PFD | 509 | PPI | 0 | Jan |
| 13 | Non-IPF | Y | N | Before | 79.4 | 60.5 | N | NIN | 486 | PPI | 0 | Apr |
| 14 | IPF | Y | N | Same | 106.8 | 42.8 | Y | NIN | 566 | PPI | 20 | Aug |
| 15 | IPF | Y | P | Before | 84.8 | 43.3 | Y | NIN | 711 | PPI | 10 | Jan |
| 16 | IPF | Y | P | Before | 58.4 | 48.9 | Y | NIN | 115 | PPI | 15 | May |
| 17 | IPF | N | P | Same | 54.9 | 23.7 | Y | NIN | 35 | PPI | 0 | Jan |
| 18 | IPF | N | P | Before | 62.6 | 27.7 | Y | NIN | 59 | PPI | 0 | Feb |
| 19 | IPF | Y | P | Same | 49 | 30.2 | Y | NIN | 21 | PPI | 15 | Feb |
| 20 | IPF | N | N | Same | 41.4 | 29.9 | Y | NIN | 18 | H2 | 5 | Feb |
| 21 | IPF | N | N | Before | 30.6 | 29.9 | Y | NIN | 106 | PPI | 20 | Aug |

SLB: surgical lung biopsy; AFA: Anti-fibrotic agent; PFD: pirfenidone; NIN: nintedanib; P: past smoker; N: never smoker; NO: no use of supplementary oxygen; Same: started oxygen at the same time as antifibrotic agent; Before: started oxygen therapy before the antifibrotic agent; RVSP: right ventricular systolic pressure; IPF: idiopathic pulmonary fibrosis; PPI: prton pump inhibitor; H2: H2 blocker; SLB: surgical lung biopsy
